# Supplementary material for: Size matters: An analysis of cigarette pack sizes across 23 European Union countries using Euromonitor data, 2006 to 2017
Source: PLoS One. 2020 Aug 13;15(8):e0237513. doi: 10.1371/journal.pone.0237513 (PMC7425903; doi:10.1371/journal.pone.0237513)
Supplement: S1 Fig — Pricing data was not available for every year in all countries (see methods for details). An expensive quartile of the market was not available for 2008 Denmark and 2016 Romania as four distinct price quartiles could not be calculated for these annual price distributions. (DOCX) [file pone.0237513.s001.docx]

S1 Fig

**S1 Fig.** Pack size category as a percentage of cheap (left-hand graphs) and expensive (right-hand graphs) quartiles based on actual

pack prices per country-year.

*Note: Pricing data was not available for every year in all countries (see methods for details). An expensive quartile of the market was not available for 2008 Denmark and 2016 Romania as four distinct price quartiles could not be calculated for these annual price distributions.*
